# The therapeutic mechanism of Compound Lurong Jiangu Capsule for the treatment of cadmium-induced osteoporosis: network pharmacology and experimental verification

**DOI:** 10.3389/fendo.2024.1331488

**Published:** 2024-07-10

**Authors:** Ya-shuang Zhou, Jian Huang, Wen-xuan Cao, Ao-xue Yu, Pan Li, Jin-ling Liang, Xiang-yang Leng, Jian Jin, Peng Yu, Jia Liu

**Affiliations:** ^1^ Changchun University of Chinese Medicine, Changchun, Jilin, China; ^2^ National Engineering Laboratory for Druggable Gene and Protein Screening, Northeast Normal University, Changchun, Jilin, China; ^3^ Medical Research Center, The Third Affiliated Hospital of Zhengzhou University, Zhengzhou, Henan, China

**Keywords:** apoptosis, Compound Lurong Jiangu Capsule, cadmium-induced osteoporosis, network pharmacology, experimental verification

## Abstract

**Background:**

Among bone diseases, osteoporosis-like skeleton, such as trabecular thinning, fracture and so on, is the main pathological change of cadmium-induced osteoporosis(Cd-OP), accompanied by brittle bone and increased fracture rate. However, the mechanism underlying cadmium-induced osteoporosis has remained elusive. Compound Lurong Jiangu Capsule (CLJC) is an experienced formula for the treatment of bone diseases, which has the effect of tonifying kidney and strengthening bones, promoting blood circulation and relieving pain.

**Objective:**

Network pharmacology and molecular docking technology combined with experiments were used to investigate the potential mechanism of CLJC in treating Cd-OP.

**Method:**

The active compounds and corresponding targets of each herb in CLJC were searched in the TCMSP and BATMAN-TCM databases. The DisGeNet, OMIM, and GeneCards databases searched for Cd-OP targets. The relationship between both of them was visualized by establishing an herb-compound-target network using Cytoscape 3.9.1 software. Gene ontology (GO), and Kyoto encyclopedia of genes and genomes (KEGG) pathway enrichment analyses were performed after determining the intersection of the targets from CLJC and Cd-OP. What’s more, molecular docking was performed to validate the results. All of them were aim to obtain hud signaling pathways for further study. Finally, BAX, BCL-2, and CASPASE-3 were screened and selected for further experiments, which included bone imaging and reconstruction analysis (Micro-CT), hematoxylin-eosin Staining (HE), and western blot (WB).

**Results:**

106 common targets from CLJC and Cd-OP targets were identified. KEGG pathway analysis suggested that multiple signaling pathways, such as the pathways in cancer, may play roles in treatment. Verification of the molecular docking was successful. Here we showed that Cd-OP displayed Tb.Th and Tb.N significantly reduced and even broke, irregular proliferation of bone cortex, uneven and loose trabecular bone arrangement, changed in apoptosis-related proteins, such as significant upregulation of CASPASE-3, BAX protein and significant downregulation of BCL-2 protein *in vivo*, while CLJC rescued these phenotypes.

**Conclusion:**

This study revealed that CLJC can reduce the expression of apoptosis-related proteins, and multiple components and multiple targets inhibit Cd-OP through apoptosis signaling pathway.

## 1 Introduction

Cadmium (Cd) is a heavy metal (1, 2) that plays an important role in productive activities. It is widely distributed in the environment and used in industry due to its good flexibility and oxidation resistance. It is essential in daily activities and production (3). However, as a highly toxic heavy metal and a serious environmental pollutant, Cd can cause toxic damage to various tissues, organs and physiological systems of humans and animals. A small amount of food consumed by animals can lead to severe poisoning symptoms, causing serious damage to a series of important organs such as liver, kidney, bone, and brain of mammals. At the same time, the functions of respiratory, digestive, nervous, reproductive, immune and other systems are damaged into human and animal bodies through direct and indirect ways (4–9). It is mainly stored in liver, kidney and bone, and its biological half-life can be as long as 10 to 30 years *in vivo*. As is known to us all, Cd induces bone metabolic diseases (10–12). Low doses of Cd induces apoptosis in osteoblasts, which are the main functional cells involved in bone formation, and lead to a decrease in bone formation (13). Under the existing circumstances of bone injury, Cd promotes the formation of osteoclasts, which are the only cells with bone resorption activity (14, 15). Importantly, Cd causes damage to both bone and osteocytes, as well as induces autophagy in osteocytes and inhibits PI3K/AKT/mTOR signaling (16). Furthermore, long-term exposure to Cd carries a high risk of bone decalcification and decrease in bone density and eventually leads to the development of fractures or osteoporosis, accompanied by pain (17, 18).

Apoptosis is one of the most common death mechanisms and plays a crucial role in the stability of the environment within a tissue, which has unique morphological and biochemical features, including cell shrinkage, chromatin condensation, and nuclear DNA degradation (19). What’s more, apoptosis is one type of programmed cell death. It is a form of single cell suicide in which cells fragmented into multiple membrane-bound particles, or apoptotic bodies, and are consumed by neighboring cells without attracting an infammatory response (20). To our surprise, in metabolic bone diseases, to control the maintenance of bone mass is quite complicated, which not only through changing in the production of osteoclasts and osteoblasts, but also through altering the duration of their respective lifespans by regulated apoptosis (21).

CLJC is an experienced formula by professor Zhang wentai of the department of orthopedics of the affiliated hospital of changchun university of traditional chinese medicine, which is a traditional chinese medicine preparation developed on the basis of the theory of traditional chinese medicine and is also a drug recommended by China Food and Drug Administration (CFDA) for the treatment of osteoporosis (22).As a prescription for bushen zhuanggu, CLJC consists of *Eucommia ulmoides, Angelica sinensis, Panaxnotoginseng, Amomum villosum Lour, Zhishouwu, Leech, Cartialgenous, Chinemys reevesii, and Dried human placenta*, which can significantly increase cortical bone thickness and trabecular bone width, improving osteoblast activity and promoting bone formation in ovariectomized (OVX) rats (23). At the same time, clinical studies showed that CLJC combined with other drugs can also increase the bone density and improve the bone metabolic index, which enhances the quality of life of patients (24). Unfortunately, the specific mechanism of CLJC for the treatment of OP has not been fully elucidated, and few experimental studies have been conducted on it. And it is challenging to explain the complex anti-osteoporosis (OP) mechanism of the herbal compound due to its multi-component and multi-target characteristics.

Network pharmacology has been applied for many years as an emerging discipline to explore and elucidate the deep mechanisms by which complex herbal remedies exert their therapeutic effects. Because its ability to establish the herbal-component-disease-target linkage, this can precisely solve the current difficulty dilemma in elucidating the mechanism of action of herbal compounding.

**Figure 1:**
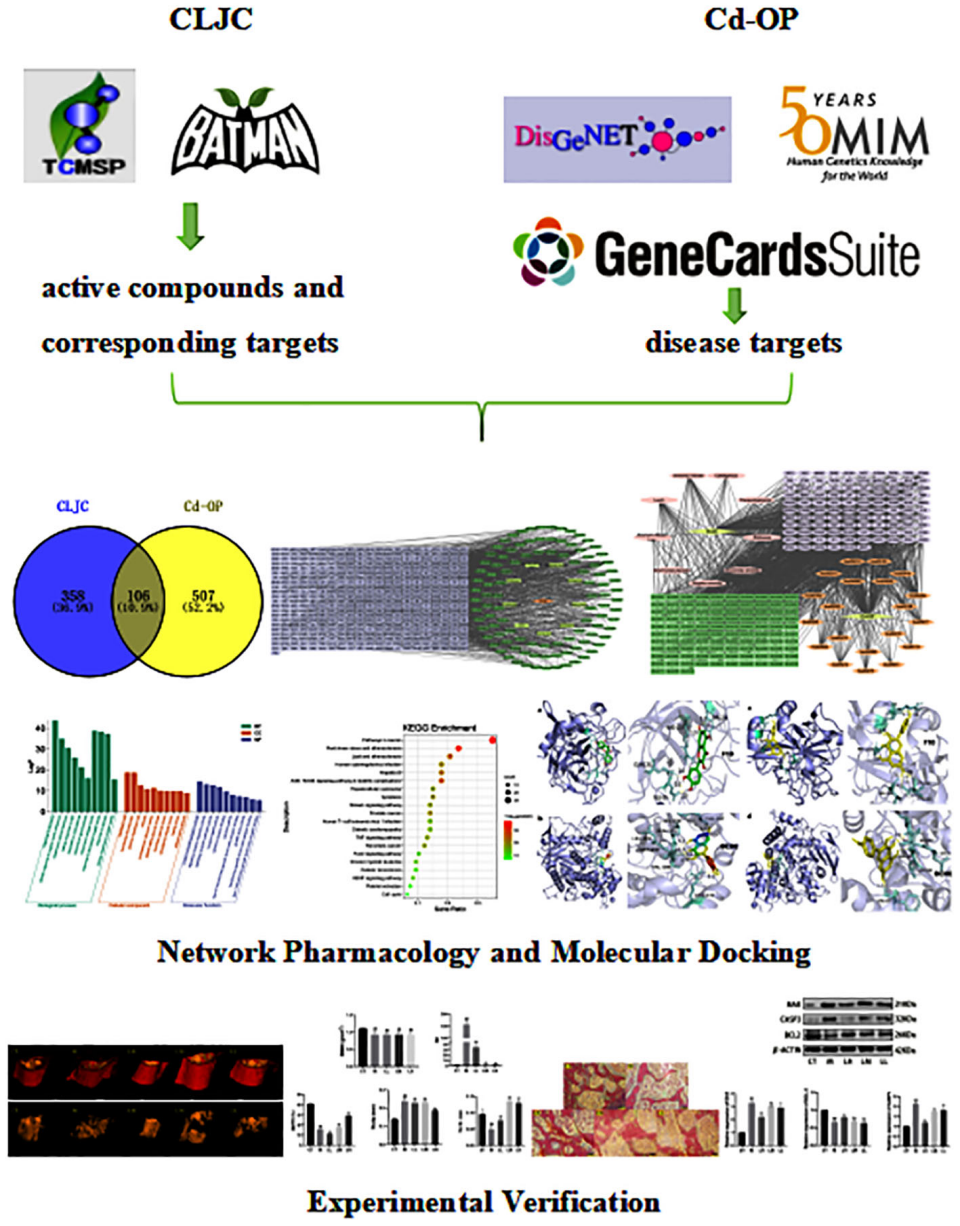
The whole article experimental design process diagram.

This study aims to investigate the hub components, targets, and pathways of CLJC for the treatment of Cd-OP through network pharmacology, molecular docking, and experimental verification (**Figure 1**), which may approach and provide a basis for further clinical application and secondary development.

## 2 Methods

### 2.1 Network pharmacology and *molecular docking*

#### 2.1.1 Compounds and *targets* of CLJC

Traditional Chinese Medicine Systems Pharmacology Database and Analysis Platform (TCMSP, https://tcmspw.com/tcmsp.php) was used to retrieve the chemical components of five herbs in CLJC, such as *Eucommia ulmoides, Angelica sinensis, Panaxnotoginseng, Amomum villosum Lour, and Zhishouwu*. At the same time, the active ingredients were screened under the filtering criteria of oral bioavailability (OB) ≥ 30% and drug-likeness (DL) ≥ 0.18. For the rest herbs of CLJC, such as *Cartialgenous, Leech, Chinemys reevesii, and Dried human placenta*, we used Bioinformatics Analysis Tool for Molecular mechANism of Traditional Chinese Medicine (BATMAN-TCM, http://bionet.ncpsb.org.cn/batman-tcm/) to collect the active ingredients of the drugs, which’s screening conditions are score cutoff ≥ 2.0 and P ≤ 0.05 as the screening conditions. Meanwhile, the corresponding target was found in the database according to the screened active ingredients. Finally, the Uniprot database (https://www.uniprot.org) was used to convert the full name of the targets to the standard gene abbreviation.

#### 2.1.2 Disease targets of Cd-OP

Using “cadmium-induced osteoporosis”,”cadmium induces osteoporosis” and “cadmium osteoporosis” separately as the search term, we searched related disease targets in the DisGeNet database (https://www.disgenet.org/), OMIM database (https://omim.org/), and GeneCards database (https://www.genecards.org/). Then, the retrieval results of the three databases were merged, the duplicate targets were deleted, and the intersection was taken as the disease target set of Cd-OP.

#### 2.1.3 Construction of Herb-Compound-Target network

To elucidate the underlying molecular mechanisms of CLJC in treating Cd-OP, a visual network of “Herb-Compound-Target” interaction was constructed. The network includes nine herbs, their corresponding active ingredients, and the intersection targets of the components and diseases. Cytoscape 3.9.1 software was used for network visualization, and topological analysis was performed based on degree values.

#### 2.1.4 Venny diagram of common targets

A Venny diagram was constructed through the online platform (https://bioinfogp.cnb.csic.es/tools/venny/) to visualize the intersection of the CLJC-related targets and Cd-OP-related targets and further clarify the potential mechanism of CLJC in treating Cd-OP.

#### 2.1.5 GO and KEGG pathway enrichment analyses

To further clarify the potential mechanism of CLJC in treating Cd-OP, we entered the common targets into the Metascape database (https://metascape.org/) for GO and KEGG pathway enrichment analyses, which can analyze molecular biological tools. At the meantime, bars and bubble maps were drawn online with bioinformatics (http://www.bioinformatics.com.cn/). The GO enrichment analysis presents biological processes (BP), cellular compositions (CC), and molecular functions (MF). And the KEGG pathway enrichment analysis shows which signaling pathways enrichment occurs.

#### 2.1.6 Construction of Herb-Compound-Target-Disease-Pathway network

To more intuitively display the entire network pharmacology analysis of CLJC in treating Cd-OP, a visual network of “Herb-Compound-Target-Disease-Pathway” interaction was constructed. The network includes nine herbs, their corresponding active compound, pathway, and the intersection targets of CLJC and Cd-OP. Cytoscape 3.9.1 software was used for network visualization. Key nodes were subsequently analyzed using the CentiScaPe2.2 plugin to screen proteins and small molecules according to the conditions that Betweenness unDir, Closeness unDir, and Degree unDir were all larger than Threshold.

#### 2.1.7 Molecular docking analysis

The screening conditions in *2.1.6* were selected for proteins and small molecules for molecular docking. The TCMSP database was searched and downloaded for small molecules. RCSB PDB database (http://www.rcsb.org/pdb/) was used to retrieve the 3D structure of the proteins and download it. Pymol 3.7 software was used for water removal to extract the 3D structure of the proteins. Pubchem database (http://pubchem.ncbi.nlm.nih.gov/) was used to retrieve and download the 3D structure of the proteins. AutodockTools1.5.7 software was applied to perform semi-flexible docking between the proteins and small molecules. The results of docking binding energy less than -5.0 kcal/mol were input to Pymol 3.7 software for visual processing.

### 2.2 Animal experiment

#### 2.2.1 Experimental animals and model establishment

6–8-week-old SPF grade C57BL/6 mice weighing from 18 to 22 g (n=5) were purchased from Liaoning Changsheng Biotechnology Co., Ltd (SCXK 2020–0001, Liaoning, China). All the animal experiments were approved by the ethics committee of changchun university of chinese medicine (ethics number 2020215). They were fed for adaptive days in the mouse feeding room of animal laboratory center of changchun university of chinese medicine for 3 days. All mice were kept inside the barrier at 23 ± 2°C of standard room temperature and 55%-70% humidity, and had free access to food and water.

After 3 days of adaptive feeding, mice were randomly divided into normal control group (CT), model control group (M), low dose of CLJC group (LL), medium dose of CLJC group (LM), and high dose of CLJC group (LH). Before the start of the model preparation, each treatment group received the corresponding dose of CLJC water solution (LL group received 0.410 g/kg CLJC by oral gavage, LM group received 0.819 g/kg CLJC by oral gavage, LH group received 1.638 g/kg CLJC by oral gavage, all drugs were determined according to the body surface area conversion method) once a day for 10 days. Both CT group and M group received equal dose of distilled water. From day 11, all mice except for CT group were intraperitoneal injected with 2.0 mg/kg cadmium chloride solution once a day for 4 weeks. During the period of mold making, CT group, M group, LL group, LM group, and LH group continued to receive the corresponding dose of distilled water and CLJC ddH_2_O solution. After the end of the experiment, the bilateral lower limb thigh muscle tissues of the mice were gently stripped, femoral samples of each group were kept as required for subsequent experiments.

#### 2.2.2 Bone imaging and reconstruction analysis

After fixation of the femur with 4% paraformaldehyde, high-resolution micro-CT scan of the distal femur with voltage 90 kV, current 80 μA, thickness 18 μm, time 14 minutes. The three-dimensional structure of the femur was reconstructed and quantified using built-in micro-CT software, including the following parameters, such as bone density (BMD), degree of anisotropy (DA), bone volume fraction (BV/TV), separation of trabecular bone (Tb.Sp), and trabecular thickness (Tb.Th).

#### 2.2.3 Hematoxylin-eosin staining

Decalcification of specimens was carried out with 10% EDTA decalcification solution (Proteintech, Wuhan Sanying Biotechnology Co., LTD, China) with constant shaking for 8 weeks. After decalcification, the specimens were embedded in paraffin and cut into 5-μm-thick sections. Three sections of the most central part of the femur were selected and underwent hematoxylin-eosin Staining.

#### 2.2.4 Western blot

Cryogenic mouse femurs were ground and split in preformed RIPA lysate and placed in a cryogenic centrifuge at 12000 g, centrifuged at 4°C for 10 minutes, and the supernatant was used to determine total protein concentration with BCA protein. Proteins were separated by polyacrylamide gel electrophoresis and transferred to a polyvinylidene fluoride (PVDF) membrane (Millipore, USA). Transprinted PVDF membranes were blocked in 5% skim dry milk for 1 hour, incubated overnight with primary antibody (1:1000), washed the next day followed by secondary antibody (1:10000) and incubated for one hour at room temperature with shaking. The main antibodies involved were BAX (1:1000, Proteintech, Wuhan, China), CASPASE-3 (1:1000, Proteintech, Wuhan, China), BCL-2 (1:1000, Proteintech, Wuhan, China), and β-actin (1:1000, CST, Boston, Massachusetts, USA). The secondary antibody was anti-rabbit immunoglobulin G (1:10000, Proteintech, Wuhan, China). Protein expression (ECL) was detected by enhanced chemiluminescence. Data analysis was performed using Image J (NIH images, Stuttgart, Germany).

### 2.3 Statistical analysis

We combined SPSS 26.0 with GraphPad Prism 8.0 software to analyze the data. The former aimed to analyze one-way analysis of variance (ANOVA) between groups, the later used to build the figures and tables. All results are presented as means ± SD, and the differences were considered crucially significant when the p-value < 0.05.

## 3 Results

### 3.1 Compounds and targets of CLJC

According to the screening rules, 171 active compounds were obtained from databases. Among these compounds, 14 were from *Cartialgenous*, 36 were from *Leech*, 30 were from *Zhishouwu*, 28 were from *Eucommia ulmoides*, 27 were from *Chinemys reevesii*, 16 were from *Dried human placenta*, 8 were from *Panaxnotoginseng*, 2 form *Angelica sinensis*, and 10 were from *Amomum villosum Lour*. 125 active ingredients were left after merging and removing the duplicates. These compounds are responsible for 1153 targets, 464 targets were left after merging and removing the duplicates.

### 3.2 Obtaining common targets of CLJC and Cd-OP

**Figure 2:**
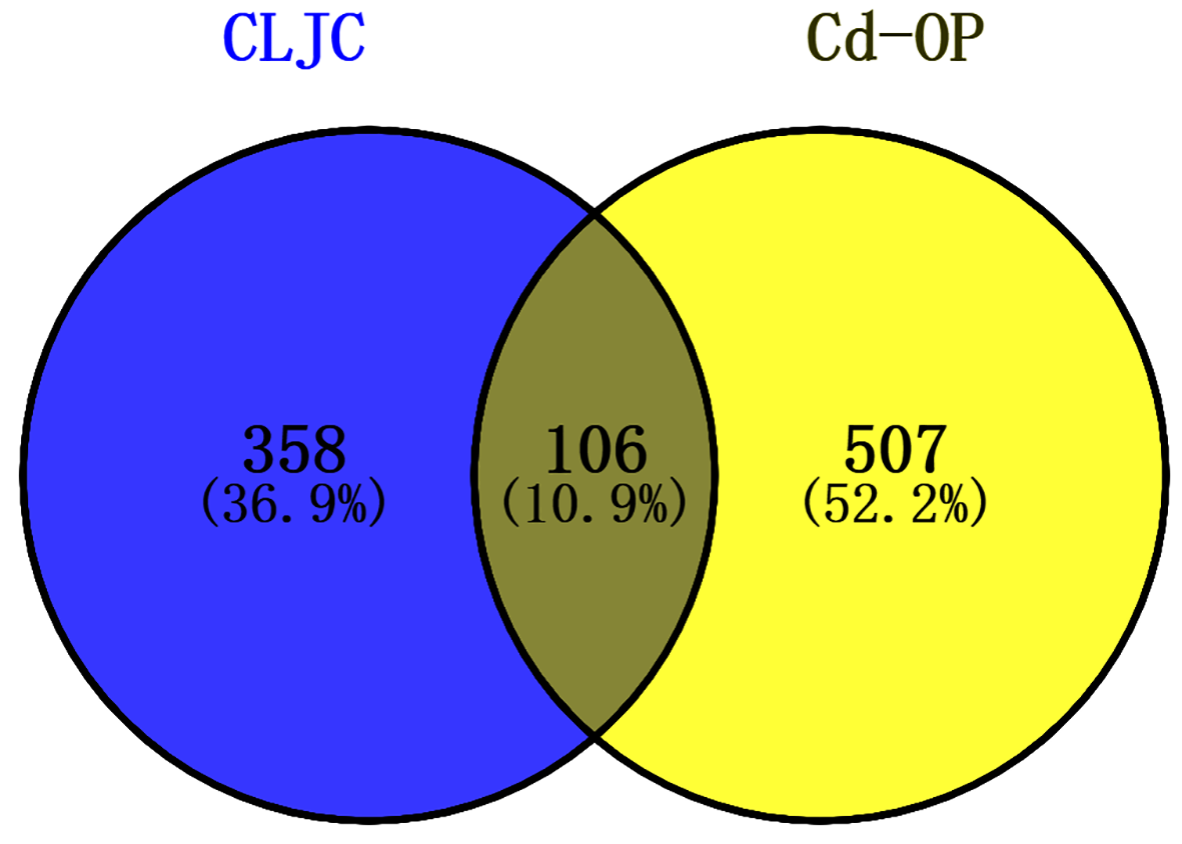
Venny diagram of CLJC and Cd-OP common targets intersection.

774 cadmium-induced osteoporosis targets were collected from GeneCards, DisGeNet, OMIM databases, 613 targets were left after merging and removing the duplicates. Venny diagram was used to identify the targets corresponding to CLJC and Cd-OP to explore the hub targets further (**Figure 2**). 106 targets were identified as common to CLJC and Cd-OP.

### 3.3 Visualization of the Herb-Compound-Target network

**Figure 3:**
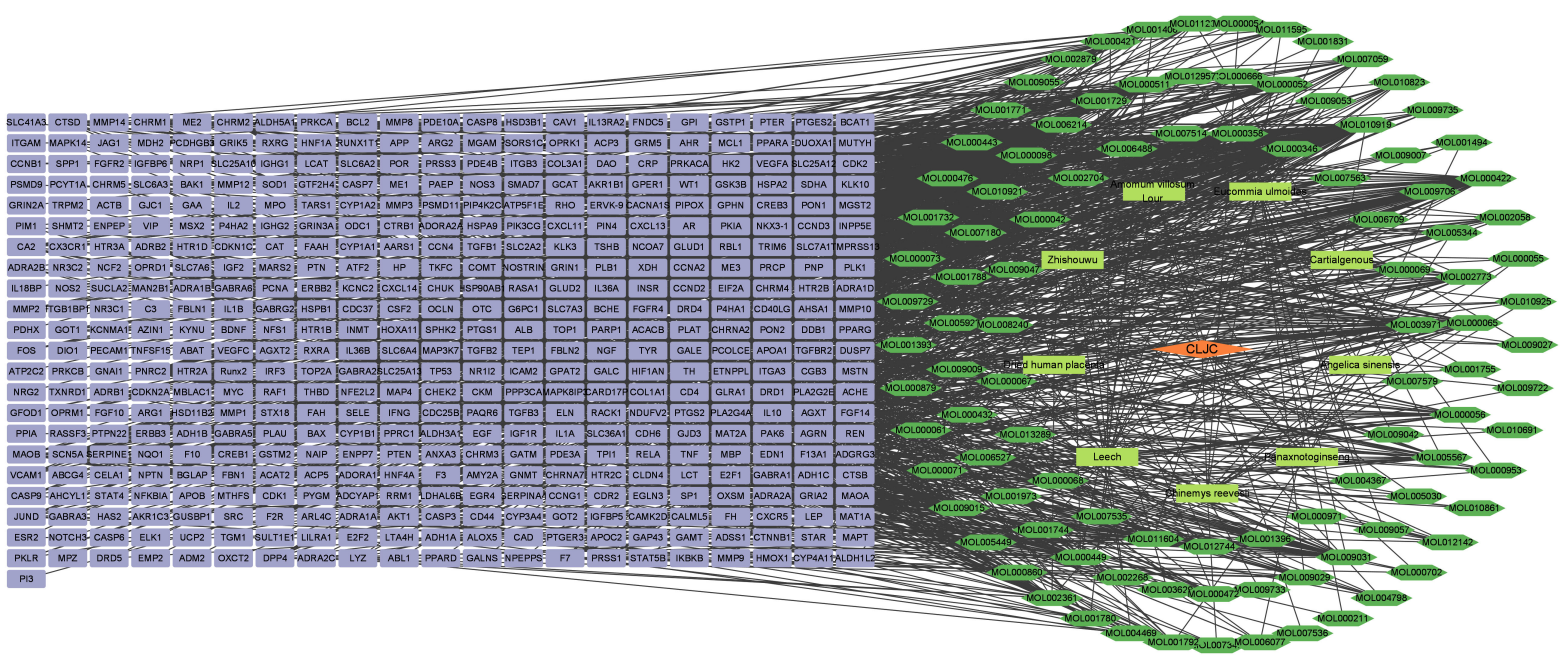
Herb-Compound-Target interaction network. Orange represents the traditional chinese medicine compound, yellow represents the compound formula, green represents the active ingredient, green represents the target.

The “Herb-Compound-Target” interaction network was constructed, including 464 targets, 125 compounds, 9 herbs. The icons of compounds were visualized (**Figure 3**). The highlighted compounds may be the essential compounds of CLJC in the treatment of Cd-OP.

### 3.4 Visualization of the GO and KEGG pathway enrichment analyses

**Figure 4:**
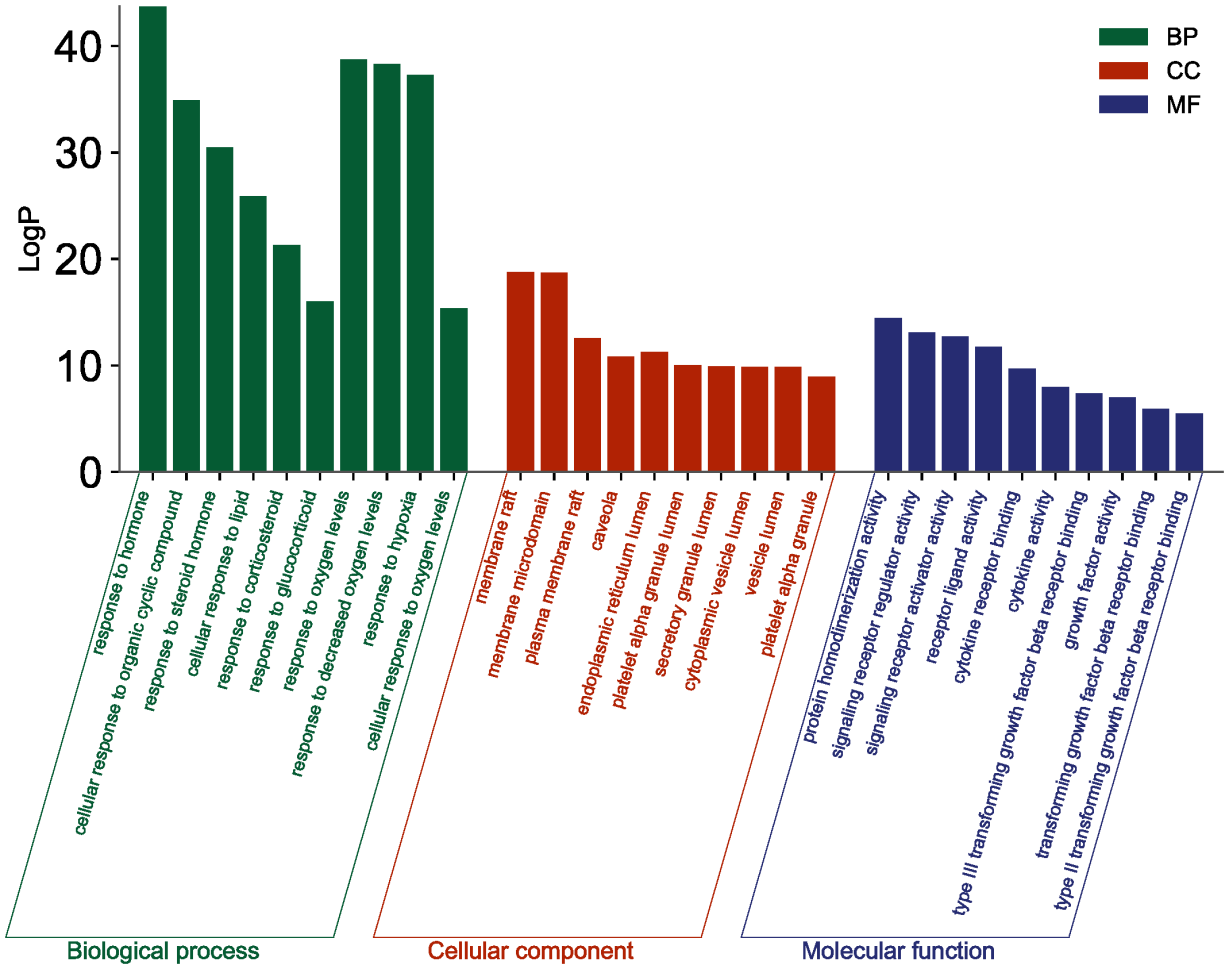
GO analysis of Cd-OP in CLJC treatment.

**Figure 5:**
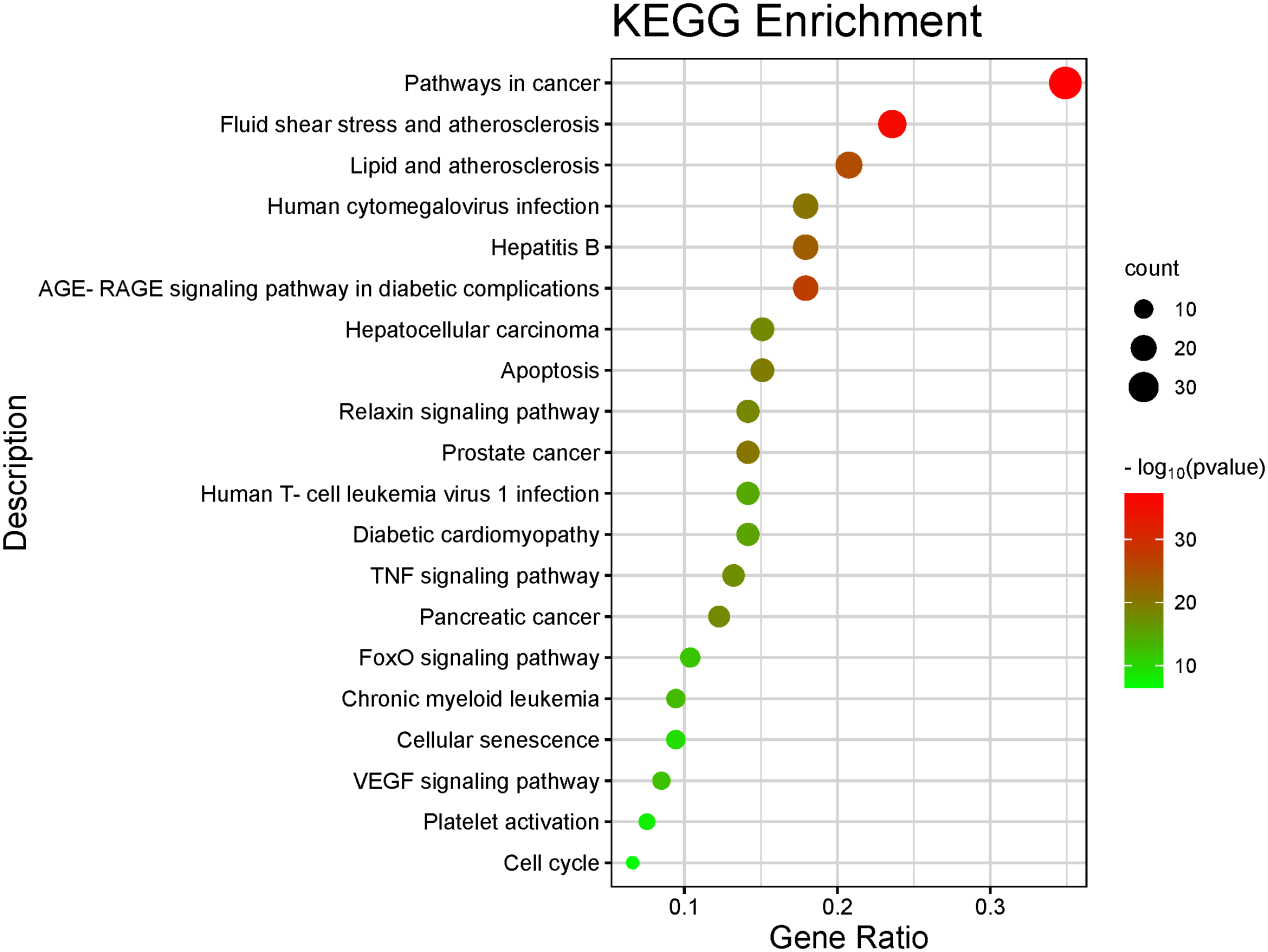
Barplot of the top 20 KEGG pathway enrichment analysis. The color of each bar represents the adjusted P value, and the size represents the number of enriched genes.

The top 10 GO and top 20 KEGG pathway enrichment analyses of CLJC for Cd-OP were extracted separately. In the GO analysis, BP have response to hormone, cellular response to organic cyclic compound, response to steroid hormone, cellular response to lipid, response to corticosteroid, response to glucocorticoid, response to oxygen levels, response to decreased oxygen levels, response to hypoxia, cellular response to oxygen levels. CC have membrane raft, membrane microdomain, plasma membrane raft, caveola, endoplasmic reticulum lumen, platelet alpha granule lumen, secretory granule lumen, cytoplasmic vesicle lumen, vesicle lumen, platelet alpha granule. MF have protein homodimerization activity, signaling receptor regulator activity, signaling receptor activator activity, receptor ligand activity, cytokine receptor binding, cytokine activity, type III transforming growth factor beta receptor binding, growth factor activity, transforming growth factor beta receptor binding, type II transforming growth factor beta receptor binding (**Figure 4**). In the top 20 pathways with the greatest significance in the 183 KEGG pathways analysis, such as pathways in cancer, pancreatic cancer, hepatocellular carcinoma, human T-cell leukemia virus 1 infection, chronic myeloid leukemia, apoptosis, were more closely connected (**Figure 5** and **Table 1**).

**Table 1:** KEGG pathways of Cd-OP in CLJC treatment.

Number	KEGG pathways	Count
Hsa05200	pathways in cancer	37
Hsa05418	fluid shear stress and atherosclerosis	25
Hsa05417	lipid and atherosclerosis	22
Hsa04933	age-rage signaling pathway in diabetic complications	19
Hsa05161	hepatitis B	19
Hsa05163	human cytomegalovirus infection	19
Hsa05225	hepatocellular carcinoma	16
Hsa04210	apoptosis	16
Hsa05166	human T-cell leukemia virus 1 infection	15
Hsa05215	prostate cancer	15
Hsa04926	relaxin signaling pathway	15
Hsa05415	diabetic cardiomyopathy	15
Hsa04668	TNF signaling pathway	14
Hsa05212	pancreatic cancer	13
Hsa04068	foxo signaling pathway	11
Hsa05220	chronic myeloid leukemia	10
Hsa04218	cellular senescence	10
Hsa04370	vegf signaling pathway	9
Hsa04611	platelet activation	8
Hsa04110	cell cycle	7

### 3.5 Construction of Herb-Compound-Target-Disease-Pathway network and molecular docking results

**Figure 6:**
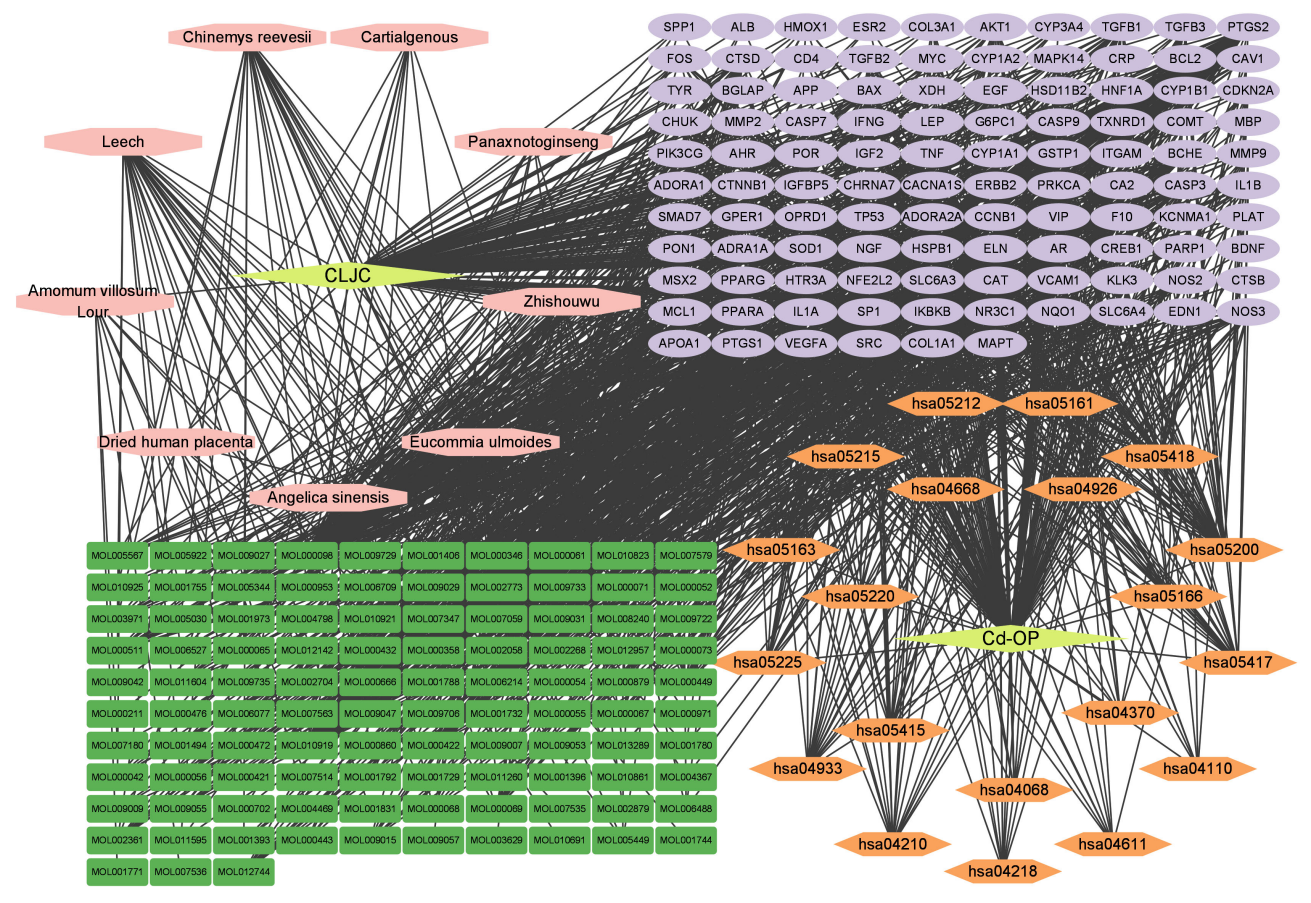
Herb-Compound-Target-Disease-Pathway network. Yellow represents the traditional chinese medicine compound and disease respectively, pink represents the compound formula, green represents the active ingredient, orange represents the pathway, and purple represents the common target.

We visualized the “Herb-Compound-Target-Disease-Pathway” network in order to more intuitively display the entire network pharmacology analysis of CLJC in treating Cd-OP. As shown in the network, components correspond to multiple targets and targets to multiple active components, indicating that CLJC induces Cd-OP through multi-component, multi-target, multi-pathway treatment of cadmium (**Figure 6**).

Network topology parameters of each node by CentiScaPe2.2 plug-in analysis, Under Betweenness unDir is greater than or equal to 383.2583, Closeness unDir is greater than or equal to 0.0016, Degree unDir is greater than or equal to 11.0833 for screening, we can conclude that the top two active ingredients (small molecules) are quercetin and kaempferol, we can also conclude that the top two targets (proteins) are F10 (Coagulation factor (F)X) and BCHE (Butyrylcholinesterase).

**Figure 7:**
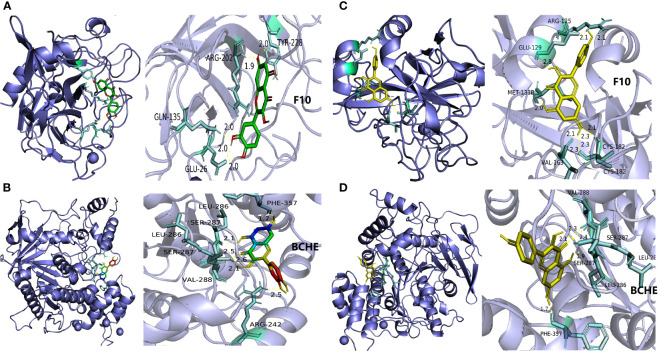
The molecular docking of quercetin and kaempferol with F10 and BCHE respectively. a is quercetin and F10, b is quercetin and BCHE, c is kaempferol and F10,d is kaempferol and BCHE.

At the same time, we used molecular docking technology for analysis to verify the binding possibility between the hub compound and the target. The docking information between the compounds and target proteins. The binding energy of F10 and kaempferol was -6.5 kcal/mol, that of BCHE and kaempferol was -7.16 kcal/mol, that of BCHE and quercetin was -6.79 kcal/mol, and that of F10 and quercetin was -6.86 kcal/mol. All the docking binding energies met the evaluation criteria of -5.0 kcal/mol, indicating that the main active components of CLJC showed better binding performance with the Cd-OP core protein targets, and the binding mode is as shown in the pictures (**Figure 7**). It can be seen from this, the quercetin and kaempferol in CLJC may act on F10 and BCHE to treat Cd-OP.

### 3.6 CLJC inhibited bone loss and alleviated bone tissue injury in Cd-OP

**Figure 8:**
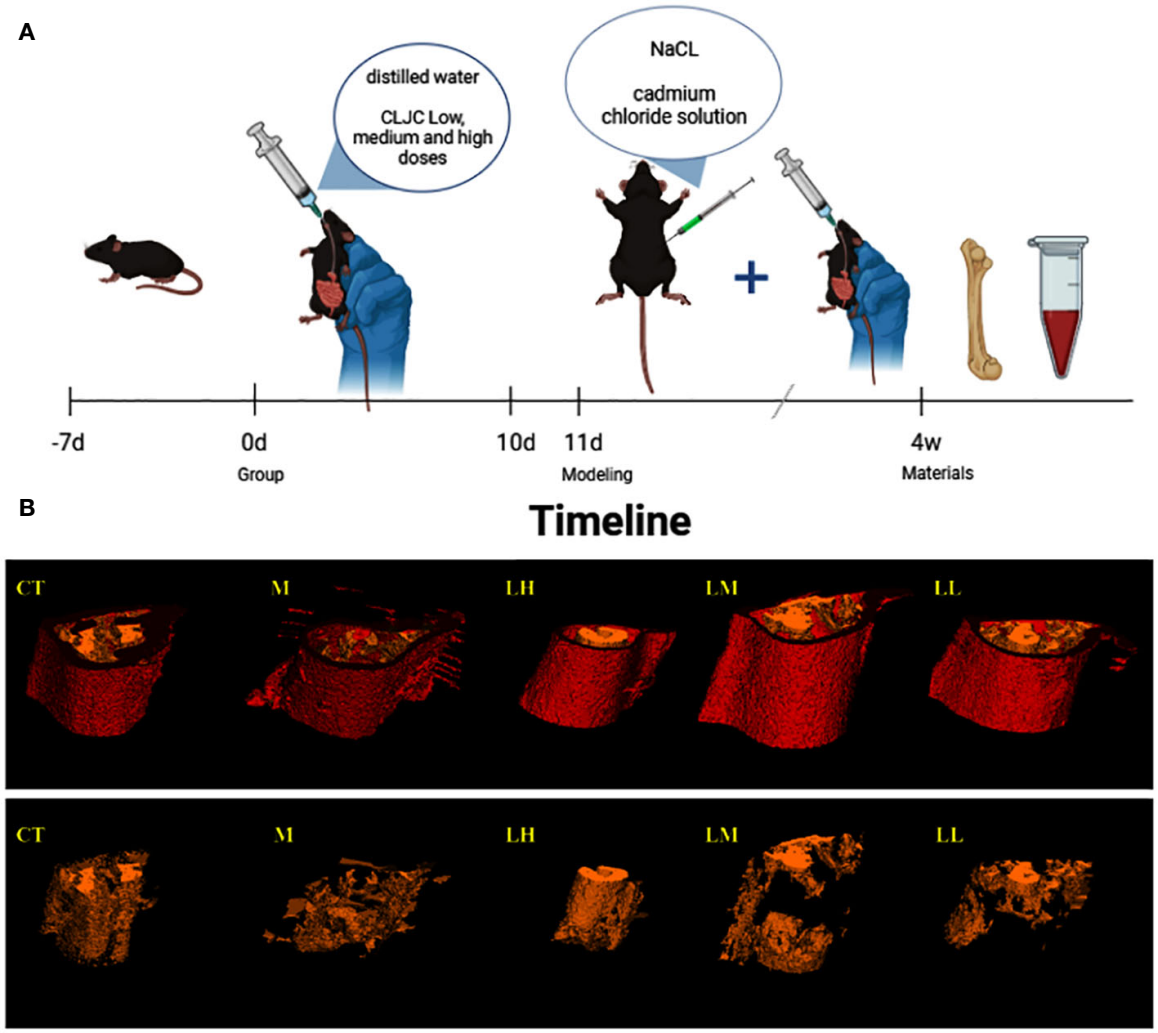
Timeline and micro-CT 3D images. **(A)** is the timeline of the entire animal experiment for a simple interpretation of the animal experiment in 2.2.1. And **(B)** is the micro-CT 3D image of the femur in each group, with red for cortex of bone and yellow for trabecular bone.

**Figure 9:**
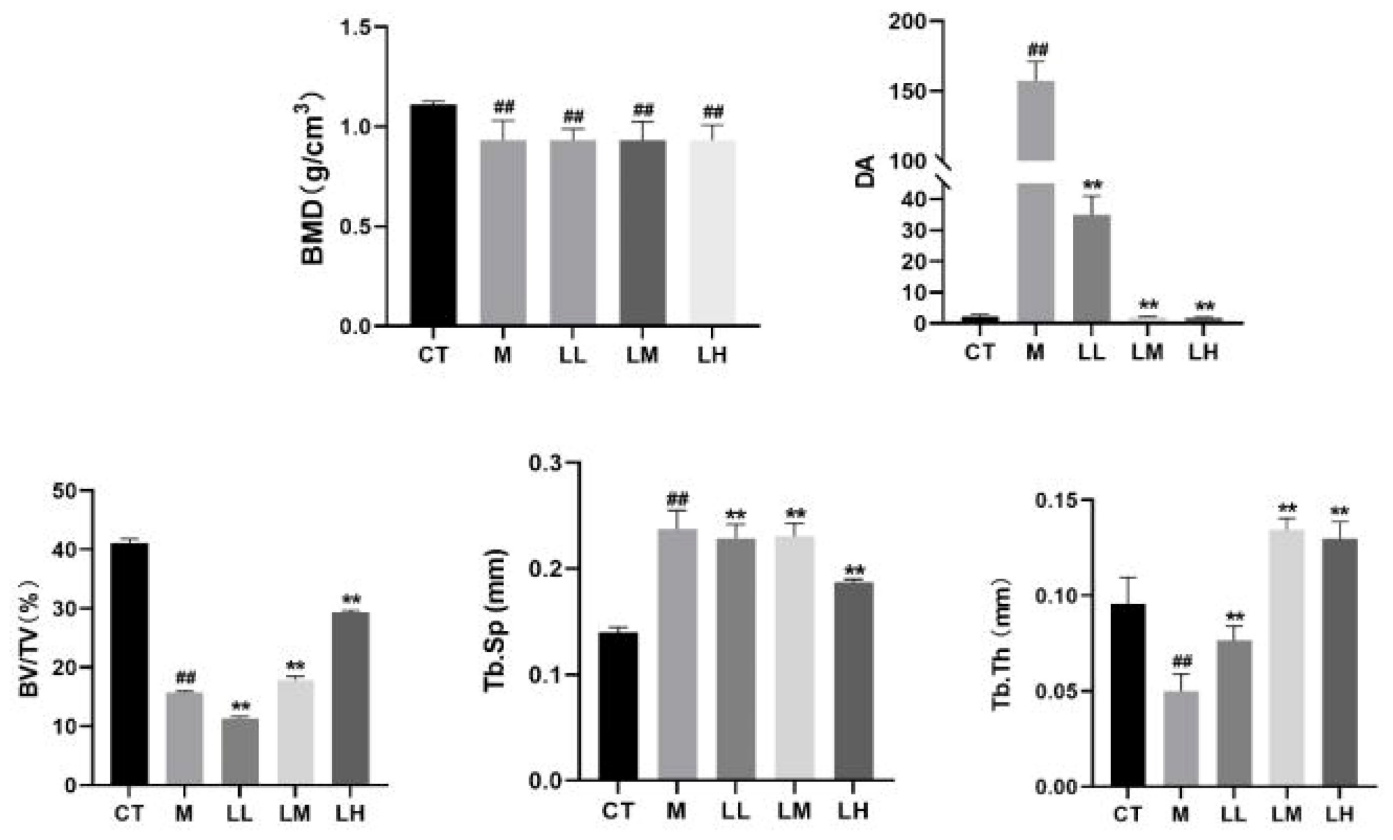
Bone histomorphometric analysis of bone density (BMD), degree of anisotropy (DA), bone volume fraction (BV/TV), separation of trabecular bone (Tb.Sp), and trabecular thickness (Tb.Th) in the trabecular bone area. ^##^p < 0.01 vs normal control group; ^★★^p < 0.01 vs model control group.

The 3D reconstruction of micro-CT indicated that there was irregular proliferation in the femoral cortex and uneven and loose trabecular bone arrangement in M group mice compared with CT group. Compared with the M group, the femoral cortex of the CLJC groups did not show significant hyperplasia, and the number of trabecular bone was relatively increased and relatively evenly distributed, among which the LH group had the best improvement effect (**Figure 8**). Bone morphometry showed that bone density (BMD) of mice in both M group and CLJC groups was significantly reduced (p<0.01).Compared with CT group, in M group, the degree of anisotropy (DA) and trabecular separation (Tb.Sp) were significantly increased (p<0.01), bone volume fraction (BV/TV) was significantly decreased (p<0.01), trabecular thickness (Tb.Th) was significantly decreased. CLJC groups mice improved to different degrees (p <0.01) (**Figure 9**).

**Figure 10:**
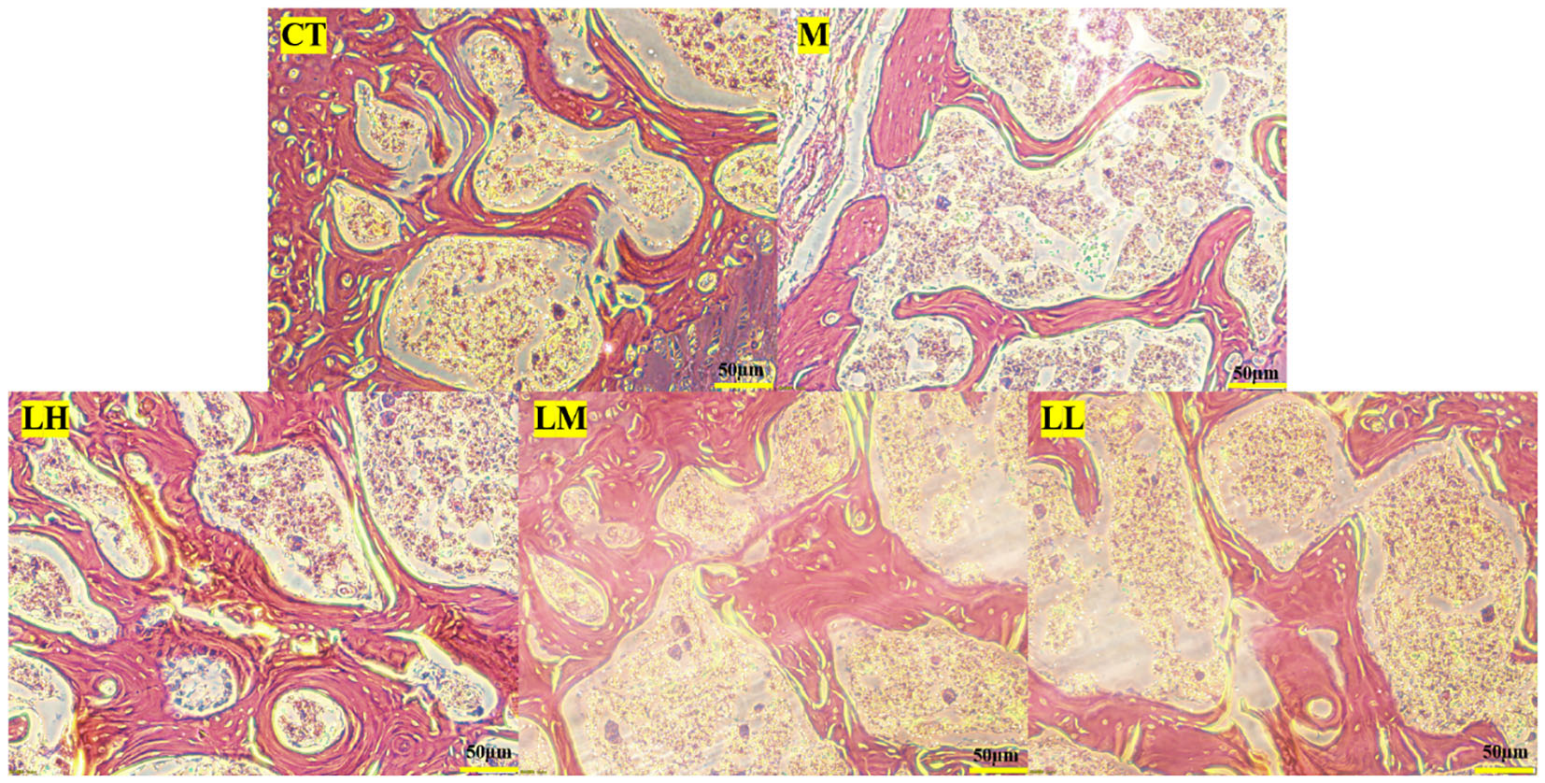
Hematoxylin-eosin staining of femur in each group.

Bone histology also demonstrated the beneficial effect of CLJC in Cd-OP. Histology of the proximal femur in M group showed thin and sparse trabecular bone, along with significant breaks. Compared with the M group, the CLJC groups had wider trabecular bone, increased trabecular bone per unit area and reduced fractures, and the LH group performed similar to the normal control group (**Figure 10**). CLJC treatment restored the architecture of trabecular bone in Cd-OP.

### 3.7 CLJC reduced CASPASE-3, BAX, and increased BCL-2 protein content in Cd-OP

**Figure 11:**
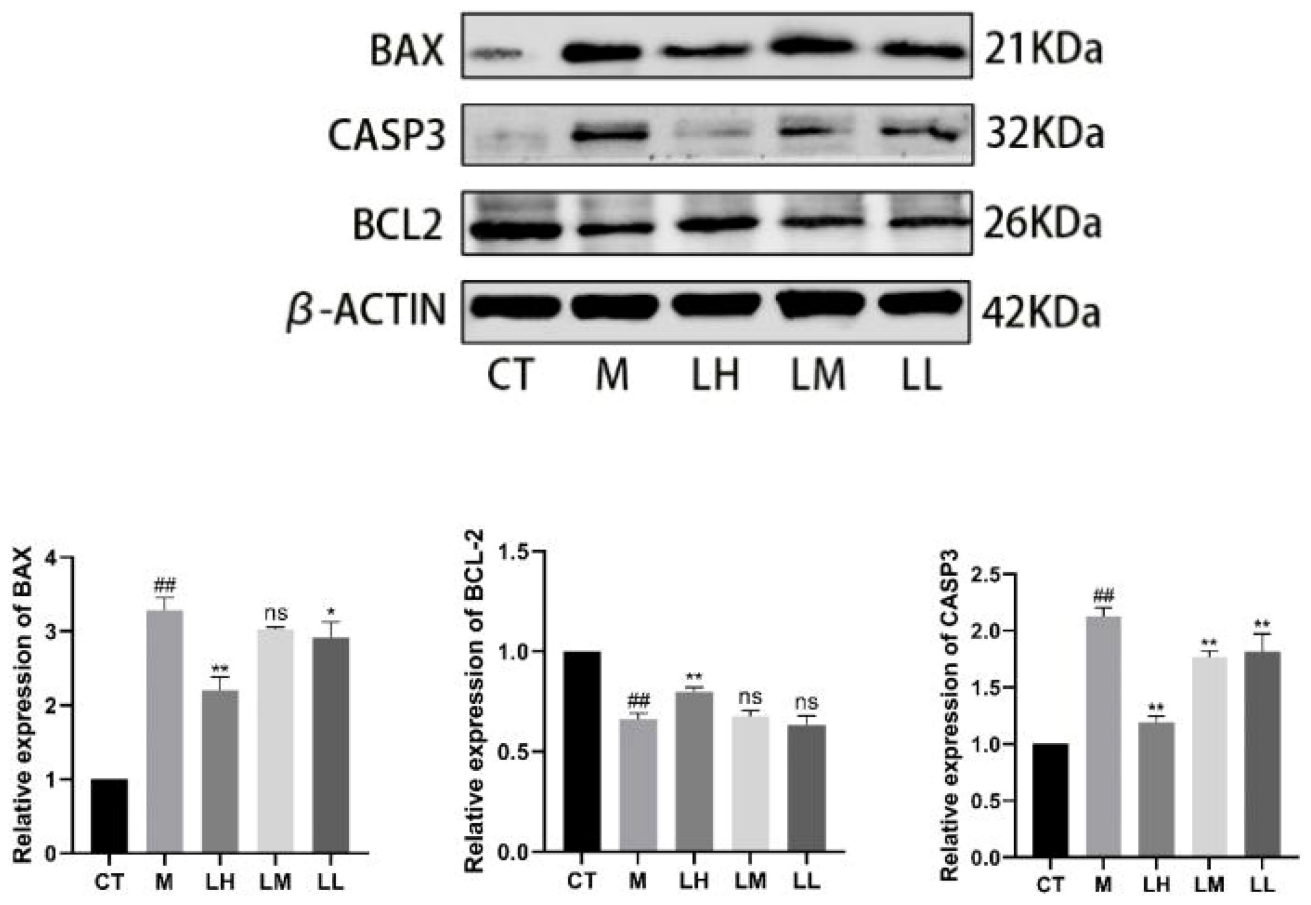
Detection of the apoptotic protein expression levels. ^##^p < 0.01 vs normal control group; ^★^p < 0.05, ^★★^p < 0.01 vs model control group; ns represents no significant difference.

Compared with the CT group, significant expression of Bcl-2 associated X protein (BAX) protein in M group (p<0.01). Compared with the M group, BAX protein was significantly down-regulated in the LH group and LL group (p <0.01, p <0.05), meanwhile, the LM group was not statistically significant compared to the M group (p> 0.05). Compared with the CT group, significant downregulation of B cell lymphoma 2 (BCL-2) protein expression in M group (p<0.01). Compared with the M group, BCL-2 protein was significantly up-regulated in the LH group (p <0.01), the LM group and LL group were not statistically significant compared with the M group (p> 0.05). Compared with the CT group, CASPASE-3 protein was significantly up-regulated in M group (p <0.01). Compared with the M group, CASPASE-3 protein was significantly down-regulated in CLJC groups (p <0.01) (**Figure 11**).

## 4 Discussion

As a metabolic bone disease, OP is influenced by other factors besides age, such as smoking, heavy metal toxicity, etc (25). Researchers reported that long-term exposure to Cd has been shown to cause bone loss, osteopenia, and osteoporosis, even increasing the incidence of fractures (26), which means that countries around the world will pay a heavy economic price for this. At the same time, considering the high cost and risk of applying the current mainstream anti-OP drugs, it is vital to explore new products from traditional medicine for the treatment of OP and to elucidate their mechanisms of action. CLJC, as a Chinese medicinal preparation, is considered by traditional chinese medicine to have the effect of supplement kidney and strong bone, invigorate blood and relieve pain, and is commonly used for symptoms, such as low back pain, lumbar, and knee weakness, and lower limb pain caused by OP (24). In China, CLJC has treated many OP patients clinically and has remarkable efficacy and exemplary safety. However, the mechanism of action is often difficult to elucidate due to the multitude of components. To research Cd-OP, we employed CLJC as the study object and used network pharmacology combinated with experiments to prove the mechanism underlying CLJC against Cd-OP and its therapeutic potential for Cd-OP.

In this study, CLJC demonstrated therapeutic efficacy for Cd-OP from both network pharmacology and animal experiment perspectives. First of all, we make use of network pharmacology approach to screen the active ingredients in CLJC, which combinated a few databases. With strictly screening and matching with differential genes of Cd-OP, we obtained 106 common targets, which were used for subsequent studies. After that, we performed GO and KEGG pathways enrichment analyses for the intersection of CLJC and Cd-OP. What’s more, we validated the molecular docking between the vital target and its corresponding compound to explore whether there is binding activity between the targets and the compounds. The results showed F10, BCHE and kaempferol, quercetin all have favorable docking activities, suggested that quercetin and kaempferol in CLJC may act on F10 and BCHE to treat Cd-OP.

Studies have shown that kaempferol can directly target mature osteoclasts and reduce osteoclast bone resorption *in vitro* by activating estrogen receptors (ER) (27). *In vivo* studies have shown that kaempferol slows glucocorticoid-induced bone loss and enhances bone strength in rats through upregulation of runt-related transcription factor 2 (RUNX2) expression levels (28). Due to its ability to bind both ERa and ERb subtypes of the ER, quercetin may be effective against OP caused by estrogen deficiency in the body, such as postmenopausal OP (29). In addition, it has been shown that quercetin slows down OP in OVX rats by inhibiting the JNK/ERK/MAPK signaling pathway and upregulating the expression level of alkaline phosphatase (ALP) (30).

Patients with OP are prone to fracture, resulting in long-term limited movement and joint braking, deep vein thrombosis of the lower limbs to block blood flow, and in severe cases, it can also lead to tissue necrosis or systemic infection. And they also have high activity of coagulation factors and are enzyme-dynamic hypercoagulable blood status (31), so the coagulation function needs to be regulated during treatment. Under the combined influence of various factors, the development of osteoporosis, and the body improves the activity of coagulation factors to prevent osteoporosis, which is a feedback protective response.

Studies proved that thrombin treatment can stimulate osteoblasts proliferation, Ca^2+^ mobilization and release prostaglandin E2, interleukin-6 (IL-6), plasminogen activator inhibitor to inhibit the differentiation and serum deprivation- or glucocorticoid-induced apoptosis of isolated osteoblasts and osteoblast-like cells (32–37). F10 is a vitamin-K-dependant plasma protein, while autocatalytic activation, activated FVII (FVIIa) converts FX into its active form FXa. In the coagulation cascade, FXa plays a significant role in converting prothrombin into active thrombin (38). FXa is not only a coagulation factor, but also like FVIIa and thrombin that induced intracellular signal transduction (39). It has been shown that FXa signals through the protease activated receptor-1 (PAR-1) to activate ERK1/2 and p38, increasing the expression of BH3-only pro-apoptotic protein Bim, leading the cleavage of caspase-3, which are the crucial markers of apoptosis (40). What’s more, FXa can lengthen phosphorylation of p42/p44 MAPK and PI3K pathways and enhance cell survival in tissue factor-stimulated baby hamster kidney (BHK) cells (41).

Bone tissue widely express several cholinergic components of the cholinergic system (transmitters, enzymes and receptors). Cholinergic activity has been shown to favor bone mass by increasing osteoblast proliferation via suppression of the sympathetic nervous system and promoting the apoptosis of osteoclasts (42, 43), playing an important role in bone remodeling (44). Therefore, inhibiting cholinergic activity can reduce bone mass, on the contrary, elevating cholinergic stimulation can reverse this situation (45). BCHE is a member of the human serine hydrolase family, an essential enzyme for cholinergic neurotransmission. It also plays significant roles in apoptosis, lipid metabolism, and xenobiotic detoxification (46). Acetylcholinesterase (ACHE) mediates osteoblast function and has an influence on bone development via cellematrix interaction (47, 48). The inhibition of the ACHE maybe reduce bone turnover by regulating bone cells proliferation and differentiation (49). The mineralization process of bone is closely related to osteoblasts in bone, and after knockout of BCHE or BCHE and ACHE, the cartilage mineralization and remodeling accelerate in ACHE (50). Other studies have shown that there is a high expression of ACHE in the apoptotic bone cells at the border of the mouse diaphysis, which is due to the pathological migration of ACHE-positive bone cells (48). Therefore, BCHE may play the same role as ACHE in different bone disease models.

Based on the above evidence, we speculate that quercetin may play an effective anti-inflammatory role by regulating cholinesterase activity, preventing the rapid degradation of acetylcholine, and maintaining the concentration of acetylcholine, it also prevented the increase of ACHE and BCHE activity induced by Cd exposure (51). In addition to many hydrophobic interactions with ACHE and BCHE, quercetin is able to bind several important amino acid residues of both enzymes, possibly one of the mechanisms that inhibit the activity of both enzymes. A hydroxyl group in the side chain is important for ACHE’s inhibitory activity. The majority of the inhibition of BCHE activity is due to the same hydroxyl group in the side chain on the B ring as compound 279, and a few to another methoxy group found on the B ring (52). Based on the above findings (53–56), we speculate that kaempferol and quercetin may exert inhibitory effects on F10 and BCHE, thus inhibiting the expression of apoptosis-related proteins, improving bone-related parameters, and exerting an effect against osteoporosis.

Meanwhile, as we all konw, it may seem to be opposing and contradictory between cell renewal and cell death, but their is no doubt that both of them are inexorably linked in bone, coupled and balanced just as tightly as are bone resorption and bone formation. Cell proliferation and apoptosis determine the prevalence and operation capacity of osteoblasts and osteoclasts commonly (21). Once osteoclasts stop resorbing bone eventually, they die by apoptosis and removed rapidly by phagocytes during the early portion of the reversal phase (57, 58), while small changes in osteoblast apoptosis can result in much larger changes in bone formation (59, 60). At the meantime, osteocyte apoptosis occurs with increasing age (61, 62). Therefore, it can be concluded that apoptosis might be the main reason for bone cell death induced by Cd, which also plays a pivotal role in Cd-induced osteoblast dysfunction, because apoptosis is an important component of bone remodeling, and abnormal apoptosis might contribute to bone loss, causing OP (63). As shown by the results of the previous KEGG pathway enrichment analysis, apoptosis signaling pathway is one of the top 20 KEGG pathways, which suggested that the apoptotic pathway may play a role in the therapeutic process. Subsequently, we performed experimental validation to investigate apoptosis-related protein changes. We measured the expression of BAX, CASPASE-3 and BCL-2 proteins. CASPASE-3 acts as an executioner in Caspase-mediated apoptosis, and the expression of CASPASE-3 positively correlates with the rate of apoptosis in cells (64). What’s more, in the BCL-2 family, BAX has a proapoptotic effect (65).We demonstrated that the M group up-regulated BAX, CASPASE-3 protein expression and down-regulated BCL-2 protein expression. Compared to the M group, the CLJC groups reversed this phenomenon, which indicated that CLJC inhibited Cd-OP to some extent.

Totally, we hypothesized that kaempferol and quercetin may play a central role in CLJC and likely play a crucial role in treating Cd-OP through apoptosis signaling pathway act on F10 and BCHE, protecting the intact development of bone cells.

## 5 Conclusion

In this study, we investigated the potential mechanism of CLJC for the treatment of Cd-OP using network pharmacology combined with molecular docking techniques, and the results showed that it acts in a multi-pathway and multi-targeted manner. We demonstrated that CLJC down-regulated the expression of BAX, and CASPASE-3 and up-regulated the expression of BCL-2 in the apoptosis signaling pathway, which might reduce apoptosis to inhibit Cd-OP. At the meantime, we hypothesized that kaempferol and quercetin may play a central role in CLJC and likely play a crucial role in treating Cd-OP through apoptosis signaling pathway act on F10 and BCHE, protecting the intact development of bone cells.

## Data availability statement

The original contributions presented in the study are included in the article/supplementary material, further inquiries can be directed to the corresponding author/s.

## Ethics statement

The animal study was approved by the ethics committee of Changchun university of chinese medicine (ethics number 2020215). The study was conducted in accordance with the local legislation and institutional requirements.

## Author contributions

YZ: Conceptualization, Data curation, Investigation, Methodology, Resources, Software, Supervision, Visualization, Writing – original draft, Writing – review & editing. JH: Conceptualization, Investigation, Project administration, Software, Validation, Writing – review & editing. WC: Investigation, Software, Supervision, Validation, Visualization, Writing – review & editing. AY: Investigation, Project administration, Resources, Software, Supervision, Writing – review & editing. PL: Data curation, Formal analysis, Investigation, Resources, Writing – review & editing. JL: Data curation, Investigation, Project administration, Resources, Writing – review & editing. XL: Formal analysis, Funding acquisition, Investigation, Methodology, Writing – review & editing. JJ: Investigation, Methodology, Project administration, Resources, Writing – review & editing. PY: Data curation, Investigation, Project administration, Resources, Writing – review & editing. JL: Investigation, Methodology, Project administration, Resources, Writing – review & editing.
